# Diabetes Cured by the Exclusive Use of Meat and Lactic Acid

**Published:** 1874-03

**Authors:** 


DIABETES CURED BY THE EXCLUSIVE USE OF MEAT AND LACTIC ACID.—This is a newly recorded case of diabetes mellitus in which Professor Cantani's mode of treatment, as above, was perfectly well borne by the patient, and produced a rapid persistent cure. The treatment did not extend beyond seventeen days. The case is recorded in Fasciculo 5 of *Lo Sperimentale*, 1873.

